# Development of a machine learning algorithm to predict the residual cognitive reserve index

**DOI:** 10.1093/braincomms/fcae240

**Published:** 2024-07-17

**Authors:** Brandon E Gavett, Sarah Tomaszewski Farias, Evan Fletcher, Keith Widaman, Rachel A Whitmer, Dan Mungas

**Affiliations:** Department of Neurology, University of California Davis School of Medicine, Sacramento, CA 95816, USA; Department of Neurology, University of California Davis School of Medicine, Sacramento, CA 95816, USA; Department of Neurology, University of California Davis School of Medicine, Sacramento, CA 95816, USA; School of Education, University of California, Riverside, Riverside, CA 92521, USA; Department of Neurology, University of California Davis School of Medicine, Sacramento, CA 95816, USA; Department of Public Health Sciences, University of California Davis, Davis, CA 95616, USA; Department of Neurology, University of California Davis School of Medicine, Sacramento, CA 95816, USA

**Keywords:** machine learning, cognitive reserve, magnetic resonance imaging, neuropsychology, aging

## Abstract

Elucidating the mechanisms by which late-life neurodegeneration causes cognitive decline requires understanding why some individuals are more resilient than others to the effects of brain change on cognition (cognitive reserve). Currently, there is no way of measuring cognitive reserve that is valid (e.g. capable of moderating brain-cognition associations), widely accessible (e.g. does not require neuroimaging and large sample sizes), and able to provide insight into resilience-promoting mechanisms. To address these limitations, this study sought to determine whether a machine learning approach to combining standard clinical variables could (i) predict a residual-based cognitive reserve criterion standard and (ii) prospectively moderate brain-cognition associations. In a training sample combining data from the University of California (UC) Davis and the Alzheimer's Disease Neuroimaging Initiative-2 (ADNI-2) cohort (*N* = 1665), we operationalized cognitive reserve using an MRI-based residual approach. An eXtreme Gradient Boosting machine learning algorithm was trained to predict this residual reserve index (RRI) using three models: Minimal (basic clinical data, such as age, education, anthropometrics, and blood pressure), Extended (Minimal model plus cognitive screening, word reading, and depression measures), and Full [Extended model plus Clinical Dementia Rating (CDR) and Everyday Cognition (ECog) scale]. External validation was performed in an independent sample of ADNI 1/3/GO participants (*N* = 1640), which examined whether the effects of brain change on cognitive change were moderated by the machine learning models’ cognitive reserve estimates. The three machine learning models differed in their accuracy and validity. The Minimal model did not correlate strongly with the criterion standard (*r* = 0.23) and did not moderate the effects of brain change on cognitive change. In contrast, the Extended and Full models were modestly correlated with the criterion standard (*r* = 0.49 and 0.54, respectively) and prospectively moderated longitudinal brain-cognition associations, outperforming other cognitive reserve proxies (education, word reading). The primary difference between the Minimal model—which did not perform well as a measure of cognitive reserve—and the Extended and Full models—which demonstrated good accuracy and validity—is the lack of cognitive performance and informant-report data in the Minimal model. This suggests that basic clinical variables like anthropometrics, vital signs, and demographics are not sufficient for estimating cognitive reserve. Rather, the most accurate and valid estimates of cognitive reserve were obtained when cognitive performance data—ideally augmented by informant-reported functioning—was used. These results indicate that a dynamic and accessible proxy for cognitive reserve can be generated for individuals without neuroimaging data and gives some insight into factors that may promote resilience.

## Teaser

Gavett and colleagues describe their use of machine learning to train models for predicting cognitive reserve. The accuracy and validity of these models were superior to conventional proxies. Predicted cognitive reserve scores, which can be generated without neuroimaging, were capable of prospectively moderating brain change effects on cognitive decline.

## Graphical Abstract

**Graphical abstract:**
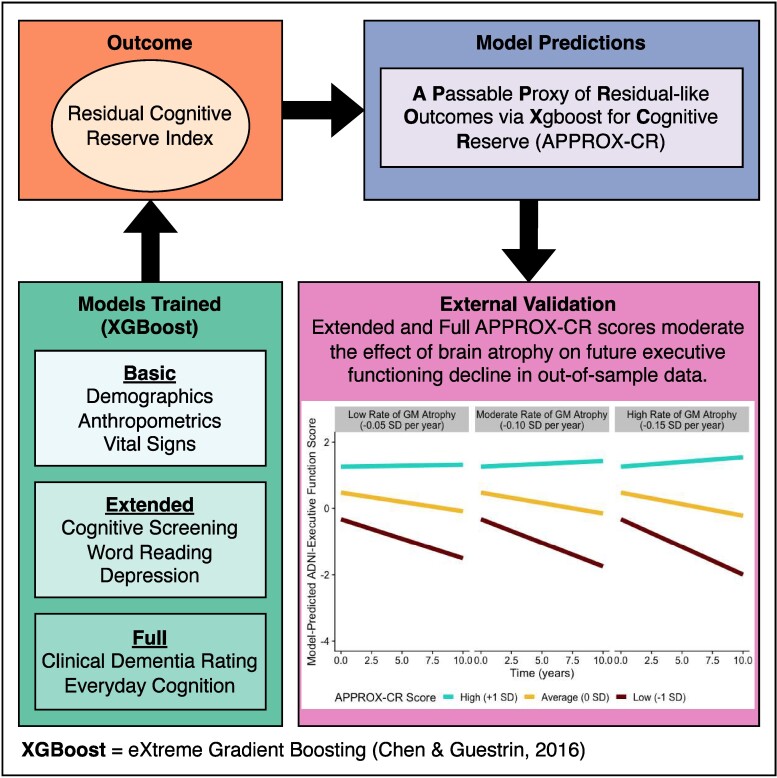

## Introduction

Gaining an improved understanding of the neural mechanisms underlying cognitive aging and dementia requires insight into individual differences in brain-cognition associations. Cognitive reserve is a construct that is intended to explain why some individuals are more resilient to age- or neuropathology-associated cognitive decline than others.^1^ Several methods have been proposed to estimate cognitive reserve. Proxy variables, like years of education and word reading ability, are the most common methods for estimating cognitive reserve, but these may be limited by their (mostly) static nature and questionable validity,^2^ among other limitations.^3^ An alternative approach to quantifying cognitive reserve was proposed by Reed and colleagues, who defined cognitive reserve as a latent variable representing the residual variance in episodic memory performance not explained by brain and demographic variables.^4^ This latent residual variance, often referred to as the residual reserve index (RRI), has been validated as a measure of cognitive reserve in numerous studies.^5-9^ Importantly, this index has been shown to prospectively moderate the effects of brain variables on cognitive outcomes, which has been described as an essential feature for establishing the validity of purported cognitive reserve markers.^10^ The RRI can also be estimated dynamically, meaning that changes in cognitive reserve over time can be captured by changes in the RRI.^11^

Despite these advantages, there are also several drawbacks to using the residual approach to estimate cognitive reserve. As discussed above, the RRI is derived by comparing observed cognitive performance to that which is expected, usually based on brain integrity and/or neuropathology. Therefore, in the absence of brain imaging data (e.g. MRI), the RRI cannot be generated, even in large research studies that may otherwise be well suited to the study of cognitive reserve. Similarly, the use of latent variable methods to estimate cognitive reserve requires a large sample of individuals with MRI or other brain imaging data, along with data pertaining to demographics and cognition, and requires the individual to undergo a neuropsychological assessment to obtain an estimate of their episodic memory ability. Therefore, use of the RRI is limited to large-scale research studies with neuroimaging and neuropsychological assessment capabilities and cannot be generated in clinical settings and applied to individual patients.

Another limitation of the RRI—although certainly not unique to this method for estimating cognitive reserve—is that it does not inherently facilitate advancements toward understanding the mechanisms that promote cognitive reserve. Unlike years of education, for example, knowing that an individual's RRI is high does not offer any insights into why it is high. This is because the RRI is, by definition, composed of unknown and/or unmeasured sources of variance that contribute to episodic memory performance.^12^ By exploring the extent to which previously ‘unmeasured’ variables can predict the RRI, we can identify candidate variables that may help elucidate the mechanisms underlying cognitive resilience to neuropathology.

In this study, we use machine learning techniques to predict cognitive reserve as measured by the RRI. Machine learning has been previously employed for prediction of cognitive reserve but in a cohort of deceased research participants who underwent autopsy.^13^ We aim to build upon this previous research by training our cognitive reserve machine learning models against an *in vivo* measure of cognitive reserve (derived using MRI data) and cross-validating them in a sample of active research participants who continued to participate in longitudinal follow-up assessments after their predicted cognitive reserve scores were generated. Such a research design has the advantage of allowing us to examine the degree to which the predicted cognitive reserve scores interact with prospective brain changes to influence longitudinal cognitive trajectories.

The goal of the current study is to develop an algorithm capable of predicting the RRI using routine, non-invasive, clinical variables that can be collected at almost any out-patient setting. There are two motivating factors underlying this goal. The first is to promote the accessibility of a valid and dynamic measure of cognitive reserve that can be applied to almost any patient or research participant. The second is to assist with the identification of variables that are most predictive of high cognitive reserve. We pursue this goal by employing machine learning algorithms trained in two large cognitive aging cohorts and cross-validating results in other non-overlapping cohorts. Three versions of the algorithm will be explored: (i) a ‘Minimal’ version, which utilizes predictor variables that can be collected at any primary care office visit or similar; (ii) an ‘Extended’ version, which augments the Minimal version with several additional patient-oriented tests and questionnaires that may be feasible to administer in most clinical settings; and (iii) a ‘Full’ version, which requires more extensive assessment plus the presence of an informant.

## Materials and methods

### Participants

The University of California (UC) Davis Alzheimer's Disease Research Center Longitudinal Diversity cohort was one source of data for this study. A second source of data was the Alzheimer's Disease Neuroimaging Initiative (ADNI) study (adni.loni.usc.edu). The ADNI was launched in 2003 as a public-private partnership, led by Principal Investigator Michael W. Weiner, MD. The primary goal of ADNI has been to test whether serial MRI, positron emission tomography, other biological markers, and clinical and neuropsychological assessment can be combined to measure the progression of mild cognitive impairment and early Alzheimer's disease.

**Figure 1:**
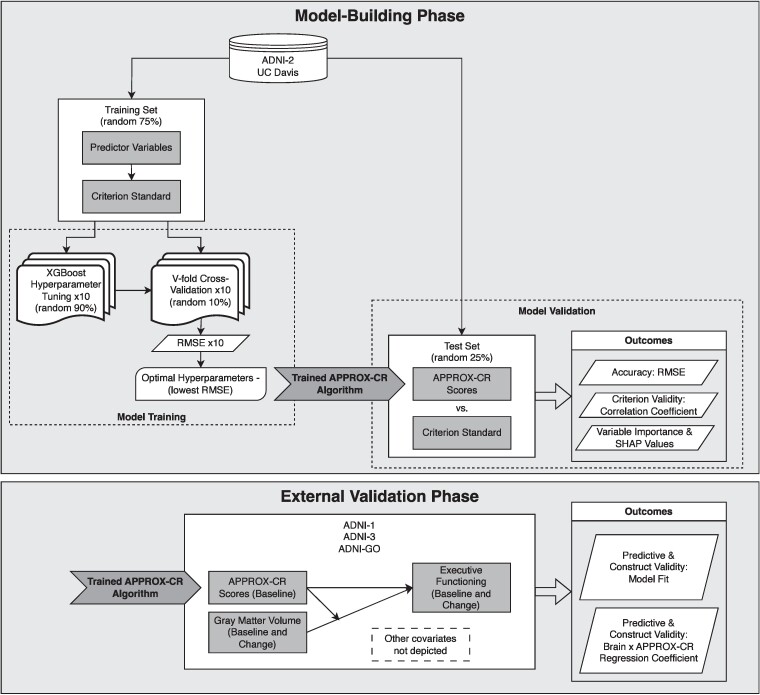
**Schematic representation of the study procedures.** In the model-building phase, the model is first trained—in a randomly-selected 75% of the combined UC Davis/ADNI-2 sample—to predict the criterion standard using XGBoost (with 10-fold cross-validation) and the trained model is subsequently validated on the 25% held-out sample. In the external validation phase, the model that was trained in the first phase was applied to an independent data set to determine whether baseline APPROX-CR scores were capable of prospectively moderating the effects of grey matter (change) on executive functioning (change). The criterion standard was the factor scores saved from the model shown in Fig. 2. The steps depicted in this figure were applied three times, once for each set of predictors (Minimal, Extended, and Full models). ADNI, Alzheimer's disease neuroimaging initiative; APPROX-CR, A passable proxy of residual-like outcomes via Xgboost for cognitive reserve; RMSE, root mean square error; SHAP, SHapley Additive explanation; UC, University of California.

This study had two phases of analysis. The first (‘model building’) phase included baseline participant data from the UC Davis cohort and the ADNI-2 cohort. In this phase, we trained a machine learning model on a randomly-selected group containing 75% of the combined UC Davis and ADNI-2 participants (training set) and used the remaining 25% (test set) to perform cross-validation of concurrent accuracy and validity. See the Supplementary material for descriptive statistics by training versus test set. The second (‘external validation’) phase included longitudinal participant data from ADNI-1, ADNI-GO, and ADNI-3. The combined external validation cohort was used to determine the predictive validity of the model in an independent data set (see the External validation subsection below for full details). A schematic diagram of the procedures used in the current study is provided in Fig. 1.

In the model-building phase, data were obtained from the visit corresponding to the first MRI scan. In contrast, the external validation phase sought to predict longitudinal cognitive outcomes, so we used all available study visits for the participants in this phase.

Details about participant recruitment into ADNI have been reported on extensively. The ADNI data used in this study were de-identified, but each ADNI site acquired written informed consent from each participant. All participants in the UC Davis cohort provided informed consent, as overseen by institutional review boards at UC at Davis, the Veterans Administration Northern California Health Care System, and San Joaquin General Hospital in Stockton, California. For more information about participant recruitment into the UC Davis cohort, see Hinton and colleagues.^14^ All human subjects research described in this study was carried out in accordance with the Declaration of Helsinki.

### Materials and methods

#### Cognitive data

The primary neuropsychological variables used in this study were composite measures of episodic memory and executive functioning. In the UC Davis cohort, episodic memory was measured using the Spanish and English Neuropsychological Assessment System (SENAS) list learning test. In the ADNI cohorts, episodic memory was measured using the ADNI-Mem composite.^15^ For external validation in ADNI-1 and ADNI-3, longitudinal ADNI-EF^16^ composite scores were used to measure executive functioning, which served as the primary outcome variable. Because the UC Davis cohort was not used for external validation, no measure of executive functioning was necessary in that sample. All of the cognitive measures used here have been well validated for use in cognitive aging research.^17-21^

#### Neuroimaging data

MRI data were obtained in each cohort. In the model-building phase, we used two neuroimaging variables: (i) a voxel-based brain grey matter signature region developed to explain as much variance in episodic memory as possible^22,23^ and (ii) white matter hyperintensity (WMH) volumes.^24^

For the external validation phase, total grey matter volume, derived using the longitudinal FreeSurfer pipeline,^25^ was obtained from the ADNI dataset for participants in ADNI-1, ADNI-GO, and ADNI-3. In particular, we extracted grey matter volume data to test the hypothesis that scores produced by our machine learning algorithm were capable of modifying the strength of association between grey matter atrophy and rate of change in executive functioning performance over time. Corrections for skull size were applied by regressing grey matter volume on intracranial volume and using the residual to capture grey matter volumes not explained by intracranial volumes.

#### Features used to predict the RRI via machine learning

##### Minimal version

The Minimal version of the machine learning algorithm was designed to use a small number of predictor variables that could be collected at almost any out-patient healthcare clinic. Predictors include age (years), years of education, sex (0 = female, 1 = male), diastolic blood pressure, pulse pressure (systolic blood pressure minus diastolic blood pressure), heart rate, height (m), body mass index (BMI, which measures weight independent of height), A Body Shape Index (ABSI, which measures waist circumference independent of height and BMI),^26^ the Hip Index (HI, which measures hip circumference independent of height, BMI, and ABSI),^27^ the Waist-Hip Index (WHI, which measures waist-to-hip ratio independent of height and BMI),^28^ and a single self-report question about whether the participant has any concerns about their memory (0 = no, 1 = yes) taken from the self-report version of the Everyday Cognition (ECog) scale.^29^ Most of the physical measurements were chosen to characterize factors relevant to metabolic disease and vascular risk. However, a polygenic risk score related to height was shown in a recent genome-wide association study to be correlated with cognitive reserve.^30^

##### Extended version

The Extended version of the machine learning algorithm added three additional predictors to the Minimal version: Mini-Mental State Examination (MMSE)^31^ or MoCA^32^ scores, American National Adult Reading Test (AMNART)^33^ scores, and Geriatric Depression Scale (GDS)^34^ scores. The MMSE and MoCA are both commonly used screening measures of global cognitive status and dementia severity that sample from domains such as orientation, memory, language, attention, and visuospatial skills, and are scored on a 30-point scale, with higher scores associated with better cognitive skills. In the UC Davis cohort, the screening measure of global cognition transitioned from the MMSE to the MoCA. To make use of all available data, MoCA scores were converted to MMSE score equivalents based on a published crosswalk study.^35^ The AMNART is a test of one's ability to pronounce orthographically irregular words. Word reading tests such as this have often been used as proxies for related constructs such as premorbid IQ, quality of education, literacy, and cognitive reserve.^36-38^ The published version of the AMNART contains 45 items. Because ADNI uses a 50-item version, we converted the 50-item AMNART scores to their 45-item equivalents using the procedures described in the Supplementary material for this manuscript. Higher scores on the AMNART are reflective of better single-word reading ability. The GDS is a 15-item true/false questionnaire about symptoms of depression that are common in older adults. Higher scores reflect more severe symptoms of depression.

##### Full version

The Full version of the machine learning algorithm incorporated additional predictors that require more time and the presence of an informant to acquire, yet are still commonly available in specialty clinics (e.g. memory clinics) or research studies. To the Extended version, the Full version adds two scores from the Clinical Dementia Rating (CDR): Sum of Boxes and the Memory Box score.^39,40^ The Full version also adds two scores from the informant version of the ECog: the total score (the average score across six domains: memory, language, visuospatial, planning, organization, and divided attention) and the individual ECog memory domain score.

### Procedure and data analysis

#### Residual reserve Index

**Figure 2:**
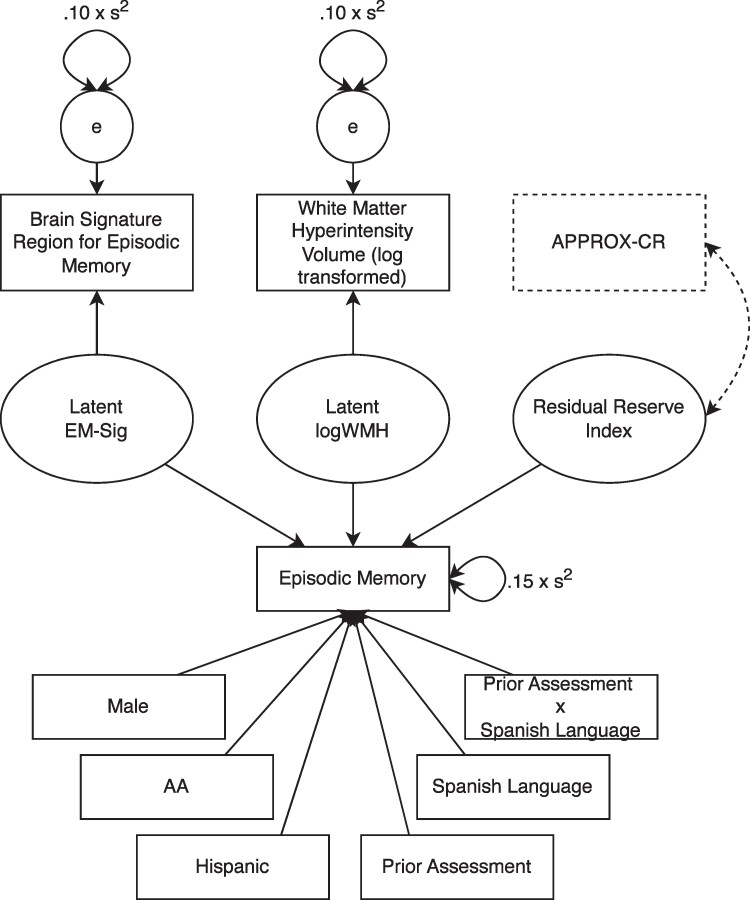
**The latent variable model used to decompose episodic memory variance and produce the residual reserve index (RRI).** Solid lines represent the model that was used to construct the RRI (the criterion standard). Dashed lines show how the model was modified to examine the concurrent validity of APPROX-CR scores (i.e. their correlation with the latent RRI). AA, African American; APPROX-CR, A passable proxy of residual-like outcomes via Xgboost for cognitive reserve; EM, episodic memory; logWMH, log-transformed white matter hyperintensity.

In the training sample, composed of UC Davis and ADNI-2 data, we constructed a latent variable model, using Mplus version 8, similar to that described by Reed *et al*.,^4^ but with several important differences. First, instead of regressing episodic memory on a formative factor composed of brain volume and hippocampus volume (both ICV-adjusted), we used a brain signature region that was specifically developed to explain as much variance as possible in episodic memory scores.^22,23^ Second, the original approach used by Reed and colleagues to construct the residual used years of education as a demographic predictor, thus making their version of the residual independent of education.^4^ In the current study, we did not regress episodic memory on education, meaning that our RRI was likely to share variance with education. This approach allowed us to treat education as a predictor of the residual and thus compare it to other potential predictors of the residual for the purpose of understanding its relative importance as a contributor to cognitive reserve. Finally, the last deviation from the Reed *et al*. model^4^ was performed to account for the fact that the UC Davis cohort included a small percentage (11.1%) of individuals who had previous exposure to neuropsychological assessment, having been enrolled in the study prior to their initial MRI scan. Therefore, in addition to regressing episodic memory performance on demographic variables (sex, race, and ethnicity), predictors of episodic memory performance also included a term to account for prior test exposure (0 = no, 1 = yes), Spanish language test administration, and a prior exposure by Spanish language interaction term to account for previous results from the UC Davis ADC cohort showing that Spanish-speaking individuals show more pronounced practice effects than English-speaking individuals.^41^ A path diagram depicting the full latent variable model used to generate the RRI is shown in Fig. 2. The components of the latent variable model depicted in solid lines were used to generate factor scores. Those saved RRI factor scores were then used as the outcome variable in the machine learning methods described next. The components of the latent variable model depicted in dashed lines were used—in a second step following the generation of factor scores—to examine the construct validity of the machine learning models.

#### Machine learning

The XGBoost (eXtreme Gradient Boosting)^42^ machine learning algorithm was used to predict the RRI factor scores. This algorithm uses a large number of decision trees, each of which is a ‘shallow’ learner. Although any single decision tree will be incapable of explaining a large proportion of the variance in the RRI, combining all of the decision trees together generates a composite prediction that is often more accurate than can be achieved using other machine learning approaches.^43,44^

The XGBoost procedure, including the tuning of model hyperparameters, was performed using the tidymodels version 1.1.0^45^ package in R version 4.3.1,^46^ with support from other packages listed in the Supplementary material for this article. The Supplementary material also provide more details about the tuned hyperparameters.

V-fold cross-validation was implemented as part of the hyperparameter tuning. A training set (a random selection of 75% of the combined UC Davis and ADNI-2 cohorts) was randomly divided into *V* = 10 different folds. Nine of these folds were combined when implementing a given combination of hyperparameters, while the 10th fold was held-out to serve as a test of the performance of a given hyperparameter set; this was repeated nine additional times so that each held-out fold could be used to determine the accuracy of the model when trained on the other nine folds. The objective function used to evaluate the accuracy with which the model could predict the RRI in the held-out folds was the root mean square error (RMSE), with the goal of minimizing this value. Hyperparameter combinations were systematically tested using the tune_bayes function. This function performs a search of *k*-dimensional hyperparameter space—where *k* is the number of hyperparameters to be tuned—using a Bayesian algorithm that attempts to predict the optimal combination of hyperparameters that will minimize the objective function RMSE given what is already known about the accuracy of prior hyperparameter combinations.^47^ We allowed the tuning algorithm to attempt as many as 200 iterations but applied an early discontinuation rule if no improvement in RMSE was found after 100 consecutive trials.

After the hyperparameter tuning was complete, the test set (the remaining 25% of the combined UC Davis and ADNI-2 cohorts that was not selected for training) was used as an additional check on the accuracy of the model. Ideally, the RMSE in the test set will not differ markedly from the RMSE in the training set. A large difference in RMSE would suggest that the XGBoost model and its chosen hyperparameters may lack generalizability to out-of-sample data. The predicted scores generated by the XGBoost algorithm are referred to here as APPROX-CR (A Passable Proxy of Residual-like Outcomes via Xgboost for Cognitive Reserve) scores.

As another check on the convergent validity of the XGBoost model, we re-ran the latent variable model described in the RRI subsection above, using only data from the test set. The model remained exactly the same, with one exception. We introduced the APPROX-CR scores into the model and requested only one parameter to be estimated: the correlation between the latent RRI and the APPROX-CR scores. All other parameters were fixed to their previously estimated values. A strong correlation between the latent RRI and the APPROX-CR scores would provide evidence for the convergent validity of the APPROX-CR scores. See the dashed lines in Fig. 2 for a depiction of how this correlation was estimated.

The steps described above were performed three times to develop the Minimal, Extended, and Full APPROX-CR algorithms.

#### External validation

Further validation of the three versions of APPROX-CR was pursued in three related cohorts, which were analyzed together: ADNI-1, ADNI-GO, and ADNI-3. The purpose of further cross-validation in these cohorts was to establish the construct validity of the APPROX-CR scores as a measure of cognitive reserve. In other words, a good proxy for cognitive reserve should do more than correlate with the criterion standard; it should also be capable of concurrently and (especially) prospectively moderating the association between brain and cognitive performance. To test this form of validity, we generated Minimal, Extended, and Full APPROX-CR scores in the ADNI-1, ADNI-GO, and ADNI-3 cohorts by submitting the clinical predictor variables to the XGBoost models established as described in the Machine Learning subsection above. We then ran a series of linear multilevel regression models that regressed executive functioning performance (i.e. ADNI-EF scores) on covariates [baseline age (centered at 70 years), years of education (centered at 12 years), sex (reference = female), and AMNART scores], grey matter volume (standardized baseline volumes and change in standardized volumes relative to baseline, measured at each time point), APPROX-CR scores, and brain-by-APPROX-CR interaction terms to test for moderation. Time was also included as a main effect and interaction term so that a test of the moderating influence of APPROX-CR scores could be applied to both the intercept and slope of executive functioning. Random effects terms for time (slope) and participant (intercept) were included in the multilevel model. For each APPROX-CR version, we used likelihood ratio tests of nested models to examine whether model fit improved relative to the baseline (covariates only) model when adding terms to examine (i) the ‘main effects’ of APPROX-CR scores on ADNI-EF, (ii) the ability of baseline APPROX-CR scores to moderate the effect of baseline brain volume on the ADNI-EF intercept, and (iii) the ability of baseline APPROX-CR scores to moderate the effects of baseline brain and brain change on ADNI-EF slopes.

## Results

Participant descriptive statistics for both samples (model building and external validation) are provided in Table 1.

**Table 1:** Participant demographics and descriptive statistics for the model-building phase and the external validation phase

	Model-building sample			External validation sample			
Variable	Overall	ADNI-2	UC Davis	Overall	ADNI-1	ADNI-3	ADNI-GO
*n*	1665	790	875	1640	819	690	131
Age, years; M (SD)	74.29 (7.47)	72.68 (7.17)	75.75 (7.44)	73.00 (7.48)	75.19 (6.84)	70.68 (7.38)	71.54 (7.88)
Education, years; M (SD)	14.65 (4.14)	16.30 (2.63)	13.16 (4.67)	15.93 (2.77)	15.53 (3.05)	16.42 (2.34)	15.82 (2.65)
Male sex; *n* (%)	780 (46.8%)	411 (52.0%)	369 (42.2%)	862 (52.6%)	477 (58.2%)	314 (45.5%)	71 (54.2%)
Race/ethnicity; *n* (%)							
Black/AA	238 (14.3%)	34 (4.3%)	204 (23.3%)	146 (8.9%)	39 (4.8%)	103 (14.9%)	4 (3.1%)
Hispanic/Latinx	236 (14.2%)	31 (3.9%)	205 (23.4%)	85 (5.2%)	19 (2.3%)	58 (8.4%)	8 (6.1%)
White/Caucasian	1151 (69.1%)	728 (92.2%)	423 (48.3%)	1414 (86.2%)	762 (93.0%)	534 (77.4%)	118 (90.1%)
Diastolic blood pressure; M (SD)	74.25 (10.38)	75.14 (9.40)	73.41 (11.18)	74.35 (9.60)	73.77 (9.57)	75.20 (9.67)	73.55 (9.22)
Pulse pressure; M (SD)	64.66 (16.99)	61.13 (14.81)	68.01 (18.22)	58.82 (14.85)	59.91 (15.37)	57.40 (14.29)	59.30 (13.89)
Heart rate; M (SD)	66.60 (10.62)	64.66 (10.31)	68.46 (10.58)	65.94 (10.74)	65.86 (10.56)	66.04 (11.05)	65.84 (10.29)
BMI; M (SD)	27.49 (5.13)	27.34 (5.19)	27.87 (4.95)	27.02 (6.20)	26.10 (4.08)	27.85 (8.01)	28.39 (5.32)
ABSI; M (SD)	80.90 (7.62)	—	80.90 (7.62)	—	—	—	—
Height, m; M (SD)	1.67 (0.10)	1.68 (0.10)	1.65 (0.10)	1.69 (0.10)	1.69 (0.10)	1.68 (0.10)	1.69 (0.11)
HI; M (SD)	104.51 (5.64)	—	104.51 (5.64)	—	—	—	—
WHI; M (SD)	3.91 (0.43)	—	3.91 (0.43)	—	—	—	—
RRI factor score; M (SD)	0.00 (1.00)	−0.17 (1.12)	0.15 (0.85)	—	—	—	—
AMNART; M (SD)	31.72 (10.12)	34.22 (8.25)	28.96 (11.21)	33.79 (9.16)	33.50 (8.97)	33.96 (9.62)	34.77 (7.82)
MMSE/MoCA; M (SD)	26.90 (3.36)	27.43 (2.72)	26.22 (3.94)	27.36 (2.62)	26.74 (2.67)	27.91 (2.54)	28.29 (1.53)
GDS; M (SD)	1.70 (2.03)	1.40 (1.41)	2.11 (2.60)	1.37 (1.46)	1.39 (1.37)	1.26 (1.54)	1.78 (1.54)
CDR-SB; M (SD)	1.53 (2.09)	1.54 (1.85)	1.52 (2.30)	1.42 (1.73)	1.80 (1.84)	1.02 (1.64)	1.24 (0.69)
CDR memory; *n* (%)							
0	664 (41.4%)	296 (37.5%)	368 (45.3%)	617 (37.6%)	230 (28.1%)	387 (56.1%)	0 (0.0%)
0.5	576 (36.0%)	314 (39.7%)	262 (32.3%)	719 (43.8%)	375 (45.8%)	216 (31.3%)	128 (97.7%)
1	322 (20.1%)	165 (20.9%)	157 (19.3%)	279 (17.0%)	199 (24.3%)	77 (11.2%)	3 (2.3%)
2	37 (2.3%)	14 (1.8%)	23 (2.8%)	25 (1.5%)	15 (1.8%)	10 (1.4%)	0 (0.0%)
3	3 (0.2%)	1 (0.1%)	2 (0.2%)	0 (0.0%)	0 (0.0%)	0 (0.0%)	0 (0.0%)
ECog (informant) memory average score; M (SD)	2.10 (0.94)	2.10 (0.94)	2.11 (0.94)	1.86 (0.88)	—	1.83 (0.90)	2.00 (0.70)
ECog (informant) total average score; M (SD)	1.76 (0.77)	1.73 (0.76)	1.79 (0.78)	1.56 (0.68)	—	1.54 (0.70)	1.62 (0.54)
ECog self-reported memory concerns; *n* (%)	611 (68.4%)	575 (74.9%)	36 (28.8%)	418 (63.7%)	—	383 (62.0%)	35 (92.1%)
Clinical diagnosis; *n* (%)							
Unavailable	2 (0.1%)	0 (0.0%)	2 (0.2%)	0 (0.0%)	0 (0.0%)	0 (0.0%)	0 (0.0%)
Dementia	287 (17.3%)	148 (18.9%)	139 (15.9%)	265 (16.3%)	193 (23.6%)	72 (10.6%)	0 (0.0%)
MCI	613 (37.0%)	341 (43.6%)	272 (31.1%)	759 (46.7%)	397 (48.5%)	234 (34.5%)	128 (99.2%)
Normal	755 (45.6%)	293 (37.5%)	462 (52.8%)	602 (37.0%)	229 (28.0%)	372 (54.9%)	1 (0.8%)

AA, African American; ABIS, A body shape index; ADNI, Alzheimer's Disease Neuroimaging Initiative; AMNART, American National Adult Reading Test; BMI, body mass index; CDR, clinical dementia rating; ECog, everyday cognition scale; GDS, geriatric depression scale; HI, hip index; MMSE, mini-mental state examination; MoCA, Montreal cognitive assessment; RRI, residual reserve index; SB, sum of boxes; WHI, waist-hip index.

— signifies that data are unavailable.

### Predictive accuracy and concurrent validity

The predictive accuracy of the models was assessed using the RMSE of the APPROX-CR scores when compared to the criterion standard: the RRI factor scores derived from the latent variable model shown in Fig. 2. In the training set, the RMSE of the Minimal, Extended, and Full APPROX-CR scores were 0.941 (SE = 0.025), 0.824 (SE = 0.008), and 0.802 (SE = 0.008), respectively. When applied to the held-out test set (25% of the combined UCD ADC and ADNI-2 cohort that was not used to train the models), the RMSE values for the Minimal, Extended, and Full versions were 1.009, 0.906, and 0.877, respectively. These results show that the model accuracy was similar in out-of-sample data, suggesting that overfitting was not a substantial problem. Both in the training and test sets, there was a sizeable improvement in accuracy between the Minimal and the Extended versions of APPROX-CR and a further, but less sizeable, improvement in accuracy for the Full version relative to the Extended version.

**Figure 3:**
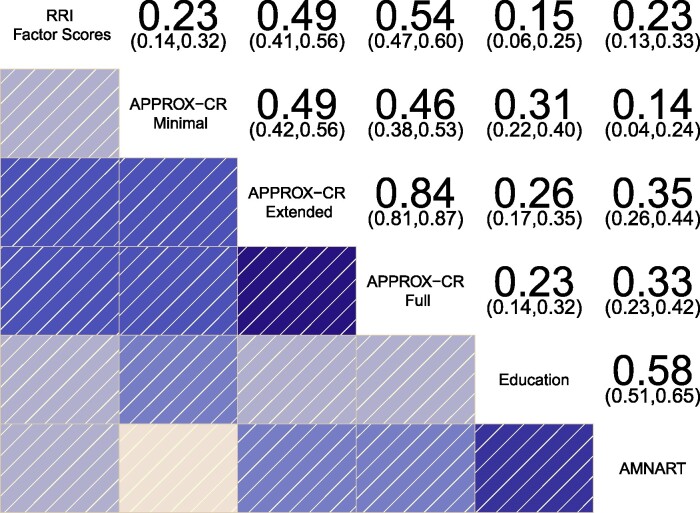
**Correlogram depicting the correlations between different methods for estimating cognitive reserve.** The residual reserve index (RRI) factor scores represent the criterion standard for evaluating concurrent validity. Numeric values represent correlation coefficients (with 95% confidence intervals), N (RRI, Minimal, Extended, Full) = 417; N (Education) = 416; N (AMNART) = 362. AMNART, American national adult reading test; APPROX-CR, A passable proxy of residual-like outcomes via Xgboost for cognitive reserve.

Concurrent validity was established by examining the degree to which the APPROX-CR scores correlated with the criterion standard in the held-out test set. Figure 3 shows a correlogram^48^ depicting the correlation matrix of the RRI factor scores (top row) with the three versions of APPROX-CR, as well as years of education and AMNART scores, for comparison purposes. The Minimal version of APPROX-CR had a relatively weak correlation with the RRI factor scores but was equivalent to the concurrent validity of the AMNART and superior to the concurrent validity of years of education (measures that have both been used individually to index cognitive reserve). A substantial improvement in concurrent validity was observed for the Extended version of APPROX-CR. Further, but less marked, improvement was seen in the Full version. These results were paralleled when using the latent RRI as the criterion standard, based on the latent variable model shown in Fig. 2. The correlations of the latent residual with the APPROX-CR scores were Minimal *r* = 0.245 (SE = 0.048), Extended *r* = 0.519 (SE = 0.037), and Full *r* = 0.574 (SE = 0.034).

### External validation

External validation applied the Minimal, Extended, and Full XGBoost models to baseline data from ADNI-1, ADNI-GO, and ADNI-3 to generate APPROX-CR scores, and then tested whether these scores were capable of moderating the effects of grey matter volume on executive functioning, after controlling for covariates. Because of differences in data availability across studies, the ADNI-1, ADNI-GO, and ADNI-3 samples did not have data for a body shape index, hip index, and waist-hip index available; these were therefore treated as missing when deriving APPROX-CR scores in the external validation sample. A series of nested model comparison tests were run to examine (i) main effects of APPROX-CR scores; (ii) the ability of APPROX-CR scores to moderate the effects of baseline grey matter volume on baseline executive functioning scores; and (iii) the ability of APPROX-CR scores to moderate the effects of baseline grey matter volume and change in grey matter volume on executive functioning performance over time.

The Minimal version of APPROX-CR did not show evidence of validity when predicting executive functioning. Relative to the covariates-only model, adding the main effects of baseline APPROX-CR scores did not lead to an improvement in fit, *χ*^2^ (df = 2) = 5.88, *P* = 0.05. Similarly, Minimal APPROX-CR scores did not moderate brain-cognition associations at baseline, *χ*^2^ (df = 1) = 0.06, *P* = 0.8, or over time, *χ*^2^ (df = 2) = 1.76, *P* = 0.42. In contrast, the Extended and Full versions of APPROX-CR showed strong validity as moderators of brain-cognition associations, both cross-sectionally and longitudinally, as seen in Table 2.

**Table 2:** Model comparisons for external validation of extended and full APPROX-CR scores

Label	Model	# Parameters	AIC	BIC	logLik	Deviance	Δχ^2^	Δdf	*P*	Comparison
Extended										
E1	Covariates Only	29	7542	7725	−3742	7484	−	−	−	−
E2	No Moderation	31	7301	7497	−3620	7239	244.8^a^	2	<0.01	E2 versus E1
E3	Intercept Moderation	32	7265	7468	−3601	7201	37.69^a^	1	<0.01	E3 versus E2
E4	Slope Moderation	34	7248	7463	−3590	7180	21.27^a^	2	<0.01	E4 versus E3
Full										
F1	Covariates Only	29	7542	7725	−3742	7484	NA	−	−	−
F2	No Moderation	31	7208	7404	−3573	7146	337.7^a^	2	<0.01	F2 versus F1
F3	Intercept Moderation	32	7157	7359	−3546	7093	53.25^a^	1	<0.01	F3 versus F2
F4	Slope Moderation	34	7126	7341	−3529	7058	35.07^a^	2	<0.01	F4 versus F3

AIC, Akaike information criterion; BIC, Bayes information criterion.

The Comparison column refers to which models (named in the Label column) are being compared with *χ*^2^ tests.

^a^ *P* < 0.01.

**Figure 4:**
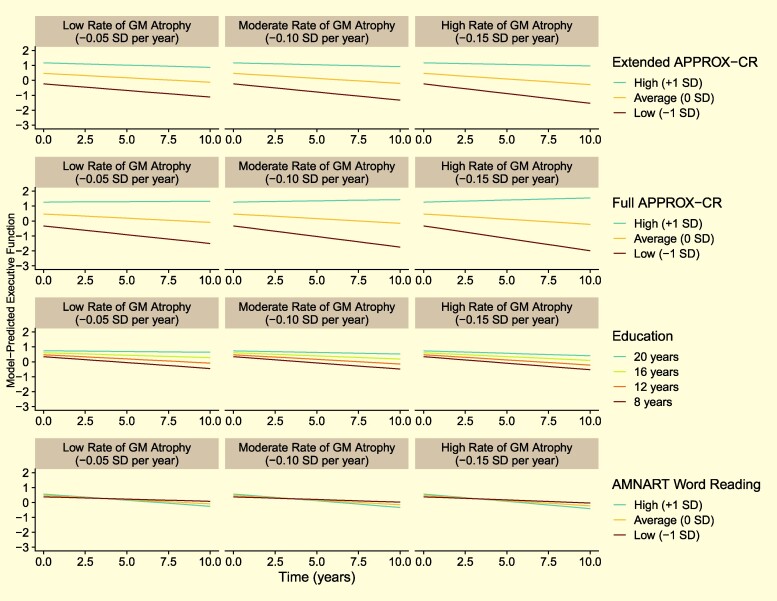
**Model-predicted trajectories for Executive Functioning performance over time for a hypothetical reference participant (female, 70 years old at baseline, 12 years of education, and with sample-average baseline brain volume).** The faceted rows compare different cognitive reserve proxies: Extended and Full APPROX-CR scores (top two rows of panels, respectively), years of education (third row of panels) and AMNART scores (bottom row of panels). The faceted columns show how the moderating influence of the cognitive reserve proxies differ by grey matter atrophy rates, ranging from less rapid (left panels) to more rapid (right panels) atrophy. GM, grey matter; MCI, mild cognitive impairment; APPROX-CR, A Passable Proxy of Residual-like Outcomes via Xgboost for Cognitive Reserve; AMNART, American National adult reading test.

Figure 4 depicts the moderating influence of Extended and Full APPROX-CR scores on executive functioning performance over time, in comparison to two other cognitive reserve proxies: years of education and AMNART scores. The results in Fig. 4 are all based on a hypothetical reference group, representing a 70-year-old woman with 12 years of education. The left column is based on sample-derived expectations for a low rate of gray matter atrophy (-0.05 SD per year). The center column is based on a moderate gray matter atrophy rate (-0.10 SD per year), and the right column is based on a high gray matter atrophy rate (-0.15 SD per year). The advantage of APPROX-CR scores—in terms of prospective moderation of brain effects on cognition—over education and AMNART scores can be appreciated visually in Fig. 4, as high APPROX-CR scores (i.e. high predicted cognitive reserve) are associated with less rapid cognitive decline, even when brain atrophy is more pronounced. For more detailed results, interested readers can obtain the parameter estimates derived from these models in the Supplementary material for this manuscript.

### Variable importance

**Figure 5:**
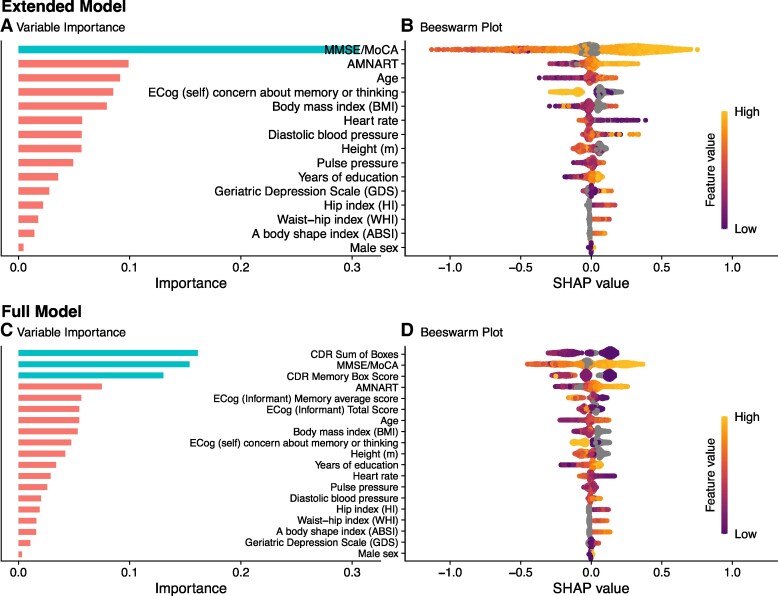
**Variable importance (left) and Beeswarm (right) plots for the extended (top) and full (bottom) versions of APPROX-CR.** The Variable Importance plots (**A** and **C**; left) rank order the features from top to bottom (*y*-axis) by their relative contribution to model predictions (*x*-axis). Color coding is used to show clusters of variables with higher (blue) versus lower (red) importance. The Beeswarm plots (**B** and **D**; right) convey how a feature's value (higher scores are more yellow, lower scores are more purple) are associated with marginal differences in predicted APPROX-CR scores (*x*-axis). When data are missing for a given feature (e.g. ABSI was not available in ADNI), feature values are colored grey. Data were derived from the training set in the Model Building sample (*N* = 1248). AMNART, American National adult reading test; CDR, clinical dementia rating; ECog, everyday cognition; MMSE, mini-mental state examination; MoCA, Montreal cognitive assessment; SHAP, SHapley additive explanation.

Figure 5 provides variable importance plots for the Extended (A) and Full (C) versions of APPROX-CR; these plots rank the relative importance of each feature used as a predictor in the Full XGBoost model. It should be noted that variable importance is a statistical phenomenon related to the quality of the predictions made, and is not necessarily reflective of importance from a mechanistic (i.e. causal) perspective. Another approach to understanding the contributions of each feature toward making predictions is through the use of SHapley Additive exPlanation (SHAP) values,^49,50^ which are shown in panels B (Extended version) and D (Full version) of Fig. 5, and also in the Supplementary material for this manuscript. The ‘beeswarm’ plots in Fig. 5B and D show the marginal impact of a given feature's value on the predicted APPROX-CR scores. For example, high MMSE/MoCA scores (colored bright yellow) are associated with higher predicted APPROX-CR values, whereas low MMSE/MoCA scores (colored dark purple) are associated with lower predicted APPROX-CR scores. Each dot represents a participant, so thicker ‘swarms’ represent more frequent occurrences in the model-building sample.

## Discussion

Current conceptualizations of cognitive reserve are important for the study of cognitive aging and dementia because they help understand and predict heterogeneity in late-life cognitive outcomes. The most easily accessible proxies for cognitive reserve, such as years of education and word reading ability, have limitations that may make them less useful than other more direct measures of cognitive reserve.^2,3^ However, more direct measures of cognitive reserve—in particular, those that use residual-based operationalizations—are less easily accessible and offer little to no insight into why some individuals have higher residual scores than other individuals. Further, neuroimaging cohorts tend to be limited by selection bias and are costly to acquire.^51^ Therefore, there is a present need to estimate cognitive reserve with the validity of residual-based models, but with the convenience of proxies.

The current study developed a machine learning algorithm to predict residual-based cognitive reserve scores, with two primary goals: (i) to provide a more easily accessible estimate of cognitive reserve that can function similarly to reserve-based methods, but without the need for cost- and time-intensive neuroimaging, comprehensive neuropsychological assessment, and with the ability to be applied to individual examinees; and (ii) to identify the variables that are most important for predicting cognitive reserve, which can facilitate future research into potentially modifiable factors that build cognitive resilience against neurodegenerative diseases of aging.^52^ Because our model, using a combination of easily accessible predictor variables, is well-correlated with the less accessible residual-based operationalization, it can be viewed as a practical bridge between the accessibility of proxy variables and the stronger validity of the residual model.

Both of this study's goals were achieved. We built three machine learning models using the XGBoost algorithm—Minimal, Extended, and Full versions—and demonstrated that the Extended and Full versions of APPROX-CR moderated the effects of grey matter volume on executive function intercept and slope. The Extended and Full versions can be applied to individual patient data to obtain an estimate of that individual's RRI had it been generated using latent variable modeling with MRI and neuropsychological performance data. Deriving the Extended APPROX-CR scores can be achieved with basic demographic, anthropometric, and physiological data, plus several brief patient-oriented screening measures (i.e. the MMSE/MoCA, AMNART, GDS, and self-reported concern about memory changes); the Full version adds the CDR and informant-report version of the ECog. Even in the presence of incomplete data, APPROX-CR scores can be derived using a simple web-based application.

The finding that the Minimal version of APPROX-CR was a poor predictor of cognitive outcomes, and was not successful in moderating the effects of grey matter volume on executive functioning performance is instructive, especially when compared to the Extended and Full versions. These latter two versions were capable of predicting cognitive intercepts and slopes, and they moderated the impact of grey matter on executive functioning, both cross-sectionally and longitudinally. In other words, the Extended and Full versions demonstrated construct validity as measures of cognitive reserve, such that high levels of reserve resulted in less rapid executive function decline, even in the context of brain atrophy (Fig. 4). The most obvious difference between the Minimal version and the more comprehensive versions was the inclusion of direct measurements of cognitive ability in the Extended and Full models. This pattern of results provides compelling evidence to suggest that predictions about an individual's current cognitive reserve should account for that individual's current level of cognitive ability. This is consistent with recent findings showing that episodic memory is a better representation of cognitive reserve compared to proxies like education,^12^ with better-moderating effects of brain-cognition associations. Stated another way, proxies that do not account for cognitive performance (e.g. years of education, occupational functioning, other life history variables) may not be successful at demonstrating the necessary feature of moderating the prospective effects of brain on cognition.^10^

In addition to possessing prospective moderation capability, APPROX-CR scores are largely dependent upon dynamic inputs; that is, variables—like cognitive performance—whose change over time during late life can be captured by repeated measurements. By including dynamic variables, APPROX-CR is more consistent with the theoretical conceptualization of cognitive reserve as a dynamic construct that could remain stable or be depleted over time, in comparison to static proxy variables like years of education.^1,9,11^ However, the Extended and Full versions of APPROX-CR also depend on education and AMNART scores, so the contributions of these (mostly) static proxy variables can be incorporated without being limited by them. Indeed, we see that in the Extended and Full versions, education and AMNART contribute some importance toward predicting outcomes, but this is greatly exceeded by dynamic cognitive and/or functional measurements (MMSE/MoCA in the Extended version and MMSE/MoCA and CDR in the Full version). As an aside, it should be noted that, while AMNART scores can change dynamically over time, these scores are usually interpreted to reflect one's premorbid IQ, which—conceptually—does not change across repeated measurements.

Examining the relative importance of each predictor in the Extended and Full models (Fig. 5) sheds some light on factors that may be most useful for estimating a person's current cognitive reserve. The sizeable improvement in performance seen in the Extended model relative to the Minimal model suggests that some direct measurement of cognition (i.e. the MMSE/MoCA and the AMNART) is highly valuable for predicting current cognitive reserve. The more modest, but still apparent, improvement in model fit of the Full version relative to the Extended version shows that more detailed information about current cognitive abilities—augmented by informant ratings of independent functioning CDR and ECog—provides additional value. When informant ratings of ECog are unavailable, an individual's self-reported memory concerns have more importance for predicting the RRI (Extended model). More specifically, in the Extended model, a self-reported cognitive concern was predictive of lower cognitive reserve (by roughly −0.1 to −0.3 standardized units), which may suggest that declining cognitive reserve may be a phenomenon capable of being subjectively experienced. Whereas previous research has demonstrated that subjective cognitive decline and associated worry about these changes is associated with worse cognitive outcomes,^53^ the current results suggest that such concerns may also be a useful predictor of cognitive reserve, especially in the absence of an informant report.

Heart rate and diastolic blood pressure had relatively greater importance in the Extended model compared to the Full model as well. This suggests that, when available, objective and informant-based measures of cognition and everyday functioning supersede physical health measures in their contribution toward predicting the RRI. Of the physical measurements, body mass index made the largest contribution to model predictions, but because the association between body mass index and predicted scores was non-linear (see the beeswarm plots in Fig. 5 and the SHAP dependence plots in the Supplementary material), this relation is likely to be nuanced and potentially moderated by other factors. Similarly, height, which was expected to show positive associations with reserve as a proxy for neurodevelopment,^30^ also demonstrated a more nuanced pattern that differed somewhat depending on the other features available in the model. Aside from height, other anthropometric and physiological measurements were toward the bottom of the feature importance scale in both the Extended and Full models, suggesting that these markers, largely associated with physical health, are less likely to be fruitful targets for research investigating the mechanisms of cognitive resilience. Notably, years of education was relatively low in importance for both the Extended and Full models. This is consistent with recent findings that static proxy measures have limited utility for characterizing cognitive reserve,^7,8,12^ especially in settings where barriers to access (e.g. educational opportunities) are inequitable. Similarly, symptoms of depression were not strong contributors toward APPROX-CR scores. It should be noted that, because XGBoost is a ‘black box’ algorithm, variables higher in importance should not be interpreted as exerting a causal influence over the outcome. However, it may be useful to consider mechanisms by which highly important variables may be predictive of cognitive reserve as an exercise in hypothesis generation for future research.

The external validation analyses directly compared APPROX-CR scores to two common cognitive reserve proxies—years of education and word reading, as measured by the AMNART—by including all three variables as moderators in the same regression model. The Extended and Full APPROX-CR scores had much stronger effect sizes when interacting with brain change to predict future rate of cognitive decline; these comparisons are depicted graphically in Fig. 4. These findings show that, even after accounting for education and word reading, having high APPROX-CR scores appears to buffer against the negative effects of brain volume change on cognitive change. It should be noted, however, that many other cognitive reserve proxies have been used in the extant literature (e.g. occupational functioning, participation in cognitively stimulating leisure activities).^54^ The current study is limited by the absence of these alternative proxies, and future research may wish to include such variables as features in a machine learning model.

Of note, the current approach to defining the criterion standard—that is, the latent RRI—used slightly different MRI measures compared to the original version described by Reed and colleagues.^4^ In the original definition of the cognitive reserve as a residual-based on the decomposition of episodic memory variance into components explained by brain variables, demographics, and the residual (i.e. what was left unexplained), hippocampal volume, total brain grey matter volume, and WMH volumes were used for the brain component.^4^ However, that method of operationalizing cognitive reserve may contain variance explained by unmeasured brain variables.^1,12,22^ To reduce the contribution of unmeasured brain to the residual, the signature region approach was used in the current study. This approach employs a data-driven search for brain substrates capable of explaining the most possible variance in an outcome of interest, and it outperforms pre-selected regions like the hippocampus.^22,23^ Thus, our use of grey matter brain signature regions was aimed at reducing the unmeasured brain component, producing a more ‘purified’ version of the residual with the goal of more accurately representing cognitive reserve.

In summary, the current results show that a residual-based proxy for cognitive reserve can be estimated using standard clinical variables that can be collected in most out-patient healthcare settings. In comparison to other readily available proxies of cognitive reserve (i.e. years of education and AMNART), APPROX-CR scores possess greater validity and, importantly, the ability to concurrently and prospectively moderate brain-cognition associations in the expected direction. In comparison to more idealized approaches to estimating cognitive reserve (i.e. a residual-based model derived from latent variable analysis of neuroimaging and neuropsychological assessment data), APPROX-CR scores are substantially more accessible, ensuring that cognitive reserve estimates can be made about almost any individual patient or research participant, even when neuroimaging facilities are unavailable.

## Supplementary Material

fcae240_Supplementary_Data

## Data Availability

ADNI data are available at https://adni.loni.usc.edu/. UC Davis data are available by request to the corresponding author, subject to a data use agreement. A web-based application for generating APPROX-CR scores, along with other analytic code used to support these analyses, can be found at https://github.com/begavett/APPROX-CR.
