# Supplementary material for: Development of a machine learning algorithm to predict the residual cognitive reserve index
Source: Brain Commun. 2024 Jul 17;6(4):fcae240. doi: 10.1093/braincomms/fcae240 (PMC11291941; doi:10.1093/braincomms/fcae240)
Supplement: fcae240_Supplementary_Data [file fcae240_supplementary_data.zip › Supplementary_material.html]

Electronic Supplement to: Development of a Machine Learning Algorithm to Predict the Residual Cognitive Reserve Index


# Electronic Supplement to: Development of a Machine Learning Algorithm to Predict the Residual Cognitive Reserve Index

#### Brandon E. Gavett1†, Sarah Tomaszewski Farias1, Evan Fletcher1, Keith Widaman2, Rachel A. Whitmer1,3, and Dan Mungas1 for the Alzheimer’s Disease Neuroimaging Initiative 1. Department of Neurology, University of California Davis School of Medicine, Sacramento, CA, USA 2. School of Education, University of California, Riverside 3. Department of Public Health Sciences, University of California Davis, Davis CA, USA † Corresponding Author Email: bgavett@ucdavis.edu (BEG)

#### 2024-06-25

# Participants

## Supplementary Table 1. Descriptive Statistics for Training and Test sets (75% and 25%, respectively, of participants sampled from the combined UC Davis and ADNI-2 cohorts)

| var | Overall | Training | Test |
| --- | --- | --- | --- |
| n | 1665 | 1248 | 417 |
| cohort = UC Davis (%) | 875 (52.6) | 632 (50.6) | 243 (58.3) |
| memr (mean (SD)) | 0.00 (1.00) | 0.01 (0.99) | -0.03 (1.04) |
| age (mean (SD)) | 74.29 (7.47) | 74.13 (7.45) | 74.79 (7.53) |
| education (mean (SD)) | 14.65 (4.14) | 14.83 (4.02) | 14.12 (4.47) |
| male = 1 (%) | 780 (46.8) | 591 (47.4) | 189 (45.3) |
| bpdias (mean (SD)) | 74.25 (10.38) | 74.20 (10.44) | 74.43 (10.22) |
| pulspres (mean (SD)) | 64.66 (16.99) | 64.41 (17.22) | 65.39 (16.28) |
| hrate (mean (SD)) | 66.60 (10.62) | 66.56 (10.43) | 66.72 (11.19) |
| bmi (mean (SD)) | 27.49 (5.13) | 27.40 (5.06) | 27.79 (5.32) |
| height\_m (mean (SD)) | 1.67 (0.10) | 1.68 (0.10) | 1.67 (0.10) |
| memcncrn = 1 (%) | 611 (68.4) | 470 (68.7) | 141 (67.5) |
| ABSI (mean (SD)) | 80.90 (7.62) | 80.90 (7.12) | 80.91 (8.76) |
| HI (mean (SD)) | 104.51 (5.64) | 104.53 (5.72) | 104.45 (5.48) |
| WHI (mean (SD)) | 3.91 (0.43) | 3.91 (0.39) | 3.91 (0.50) |
| amnart45 (mean (SD)) | 31.72 (10.12) | 31.79 (10.01) | 31.50 (10.45) |
| adj\_mmse (mean (SD)) | 26.90 (3.36) | 26.96 (3.35) | 26.74 (3.40) |
| gds (mean (SD)) | 1.70 (2.03) | 1.68 (1.99) | 1.75 (2.15) |
| cdrsum (mean (SD)) | 1.53 (2.09) | 1.55 (2.12) | 1.48 (2.02) |
| memory (%) |  |  |  |
| 0 | 664 (41.4) | 506 (42.1) | 158 (39.4) |
| 0.5 | 576 (36.0) | 417 (34.7) | 159 (39.7) |
| 1 | 322 (20.1) | 248 (20.6) | 74 (18.5) |
| 2 | 37 ( 2.3) | 27 ( 2.2) | 10 ( 2.5) |
| 3 | 3 ( 0.2) | 3 ( 0.2) | 0 ( 0.0) |
| ecogmem (mean (SD)) | 2.10 (0.94) | 2.11 (0.94) | 2.09 (0.94) |
| ecog\_avg (mean (SD)) | 1.76 (0.77) | 1.75 (0.77) | 1.77 (0.77) |

# Procedure

## AMNART

Because ADNI uses a 50-item version of the AMNART, which is different
from the published 45-item version that is used in the UC Davis cohort,
we needed to convert the 50-item AMNART scores to 45-item equivalents.
Fortunately, item-level AMNART data is available in ADNI, but only in
the ADNI-1 cohort. That means a 45-item version can’t be obtained from
the item-level data for other ADNI participants. Therefore, we needed to
convert 50-item scores to their 45-item equivalents. To do this, we
applied item response theory (2 parameter logistic model) using the
`mirt` package version 1.41.8 to estimate difficulty and
discrimination parameters for each of the 50 binary items in ADNI-1.
Once each item’s parameters had been estimated, we used those parameters
to generate expected test scores for both versions – 50 item and 45 item
– for all true ability levels (theta) ranging from 6 SD below the mean
to 6 SD above the mean (see Panel A of Supplementary Figure 1). As a
result, every 50-item AMNART score had a 45-item equivalent, and both
were derived from the same underlying ability level. We regressed the
expected 45-item AMNART scores on expected 50-item AMNART scores – while
including quadratic and cubic terms in the model to account for
non-linear associations – to allow us to predict an expected 45-item
AMNART score for ADNI participants from other cohorts (i.e., ADNI-2,
ADNI-GO, ADNI-3) where only total 50-item AMNART scores were available.
See panel B of Supplementary Figure 1. Based on this regression model,
we created a lookup table to make score conversions, which is shown
below.

### Supplementary Table 2. IRT Parameter Estimates for the AMNART

|  | a | b | g | u |
| --- | --- | --- | --- | --- |
| **ANART\_Q1** | 2.335 | -2.216 | 0 | 1 |
| **ANART\_Q2** | 1.829 | -2.087 | 0 | 1 |
| **ANART\_Q3** | 1.44 | -2.687 | 0 | 1 |
| **ANART\_Q4** | 0.6029 | -4.417 | 0 | 1 |
| **ANART\_Q5** | 2.152 | -2.438 | 0 | 1 |
| **ANART\_Q6** | 1.584 | -2.088 | 0 | 1 |
| **ANART\_Q7** | 2.016 | -1.902 | 0 | 1 |
| **ANART\_Q8** | 1.309 | -1.741 | 0 | 1 |
| **ANART\_Q9** | 2.141 | -1.666 | 0 | 1 |
| **ANART\_Q10** | 3.686 | -2.191 | 0 | 1 |
| **ANART\_Q11** | 2.883 | -1.339 | 0 | 1 |
| **ANART\_Q12** | 2.253 | -2.075 | 0 | 1 |
| **ANART\_Q13** | 1.517 | -2.598 | 0 | 1 |
| **ANART\_Q14** | 2.163 | -1.583 | 0 | 1 |
| **ANART\_Q15** | 1.5 | -0.5956 | 0 | 1 |
| **ANART\_Q16** | 1.879 | -2.239 | 0 | 1 |
| **ANART\_Q17** | 0.7363 | -2.603 | 0 | 1 |
| **ANART\_Q18** | 1.724 | -2.001 | 0 | 1 |
| **ANART\_Q19** | 1.475 | -0.1807 | 0 | 1 |
| **ANART\_Q20** | 2.073 | -0.4211 | 0 | 1 |
| **ANART\_Q21** | 2.199 | -0.6469 | 0 | 1 |
| **ANART\_Q22** | 1.433 | -0.46 | 0 | 1 |
| **ANART\_Q23** | 1.604 | -0.7292 | 0 | 1 |
| **ANART\_Q24** | 1.681 | -2.591 | 0 | 1 |
| **ANART\_Q25** | 1.643 | -1.164 | 0 | 1 |
| **ANART\_Q26** | 2.051 | -0.8549 | 0 | 1 |
| **ANART\_Q27** | 2.504 | -1.397 | 0 | 1 |
| **ANART\_Q28** | 1.952 | -0.5126 | 0 | 1 |
| **ANART\_Q29** | 1.349 | -1.285 | 0 | 1 |
| **ANART\_Q30** | 1.182 | 0.2039 | 0 | 1 |
| **ANART\_Q31** | 1.715 | -1.303 | 0 | 1 |
| **ANART\_Q32** | 2.112 | -1.36 | 0 | 1 |
| **ANART\_Q33** | 2.401 | -0.6836 | 0 | 1 |
| **ANART\_Q34** | 2.004 | -0.6764 | 0 | 1 |
| **ANART\_Q35** | 1.45 | -1.119 | 0 | 1 |
| **ANART\_Q36** | 1.51 | -1.694 | 0 | 1 |
| **ANART\_Q37** | 2.017 | -0.05772 | 0 | 1 |
| **ANART\_Q38** | 1.079 | -0.4842 | 0 | 1 |
| **ANART\_Q39** | 1.548 | -1.086 | 0 | 1 |
| **ANART\_Q40** | 1.26 | -0.9017 | 0 | 1 |
| **ANART\_Q41** | 1.025 | 0.2392 | 0 | 1 |
| **ANART\_Q42** | 1.405 | 0.2064 | 0 | 1 |
| **ANART\_Q43** | 1.148 | -0.5225 | 0 | 1 |
| **ANART\_Q44** | 2.443 | -0.2517 | 0 | 1 |
| **ANART\_Q45** | 1.581 | -1.912 | 0 | 1 |
| **ANART\_Q46** | 1.641 | 1.494 | 0 | 1 |
| **ANART\_Q47** | 1.427 | 0.6394 | 0 | 1 |
| **ANART\_Q48** | 2.196 | -0.09581 | 0 | 1 |
| **ANART\_Q49** | 1.193 | 0.9395 | 0 | 1 |
| **ANART\_Q50** | 1.366 | -0.1506 | 0 | 1 |

### Supplementary Figure 1. Association Between AMNART45 and AMNART50

Supplementary Figure 1. Association Between AMNART45 and AMNART50. Panel
A (left) shows the associations between true word reading ability
(Theta; x-axis) and expected test score (y-axis) for the 45- and 50-item
AMNART versions (red and blue lines, respectively). Panel B (right)
shows the crosswalk between the 50-item AMNART score (x-axis) and the
45-item AMNART score (y-axis). AMNART = American National Adult Reading
Test.

### Supplementary Table 3. Crosswalk between 50-item and 45-item AMNART

| amnart\_50 | amnart\_45 |
| --- | --- |
| 0 | 0 |
| 1 | 0.7953 |
| 2 | 1.601 |
| 3 | 2.418 |
| 4 | 3.244 |
| 5 | 4.08 |
| 6 | 4.925 |
| 7 | 5.779 |
| 8 | 6.641 |
| 9 | 7.511 |
| 10 | 8.388 |
| 11 | 9.273 |
| 12 | 10.16 |
| 13 | 11.06 |
| 14 | 11.97 |
| 15 | 12.88 |
| 16 | 13.79 |
| 17 | 14.71 |
| 18 | 15.63 |
| 19 | 16.56 |
| 20 | 17.49 |
| 21 | 18.42 |
| 22 | 19.36 |
| 23 | 20.3 |
| 24 | 21.24 |
| 25 | 22.18 |
| 26 | 23.13 |
| 27 | 24.07 |
| 28 | 25.02 |
| 29 | 25.96 |
| 30 | 26.91 |
| 31 | 27.85 |
| 32 | 28.79 |
| 33 | 29.73 |
| 34 | 30.67 |
| 35 | 31.61 |
| 36 | 32.54 |
| 37 | 33.47 |
| 38 | 34.4 |
| 39 | 35.32 |
| 40 | 36.24 |
| 41 | 37.15 |
| 42 | 38.06 |
| 43 | 38.96 |
| 44 | 39.85 |
| 45 | 40.74 |
| 46 | 41.63 |
| 47 | 42.5 |
| 48 | 43.37 |
| 49 | 44.23 |
| 50 | 45 |

# Results

## Supplementary Table 4. XGBoost Model Hyperparameters

| Version | mtry | trees | min\_n | tree\_depth | learn\_rate | loss\_reduction | sample\_size |
| --- | --- | --- | --- | --- | --- | --- | --- |
| Minimal | 6 | 1211 | 21 | 1 | 0.01609 | 3.843e-10 | 0.2512 |
| Extended | 17 | 574 | 3 | 4 | 0.005986 | 9.181e-06 | 0.4862 |
| Full | 7 | 1481 | 21 | 3 | 0.003432 | 0.004626 | 0.5374 |

## Supplementary Figure 2. Feature Importance

Supplementary Figure 2. Feature importance plots for the Minimal (top),
Extended (middle), and Full (bottom) XGBoost models for predicting the
residual reserve index factor scores. ECog = Everyday Cognition; MMSE =
Mini-Mental State Examination; MoCA = Montreal Cognitive Assessment;
AMNART = American National Adult Reading Test; CDR = Clinical Dementia
Rating.

## Supplementary Figure 3. Concurrent Validity

Supplementary Figure 3. Correlogram depicting the correlations between
different methods for estimating cognitive reserve. The residual reserve
index (RRI) factor scores represent the criterion standard for
evaluating concurrent validity. Numeric values represent correlation
coefficients (with 95% confidence intervals), N (RRI, Minimal, Extended,
Full) = 417; N (Education) = 416; N (AMNART) = 362. APPROX-CR = A
Passable Proxy of Residual-like Outcomes via Xgboost for Cognitive
Reserve; AMNART = American National Adult Reading Test.

## Model Comparisons

### Supplementary Table 5. Minimal Version

| npar | AIC | BIC | logLik | deviance | Chisq | Df | p |
| --- | --- | --- | --- | --- | --- | --- | --- |
| 29 | 7542 | 7725 | -3742 | 7484 | NA | NA | NA |
| 31 | 7540 | 7736 | -3739 | 7478 | 5.884 | 2 | 0.05277 |
| 32 | 7542 | 7744 | -3739 | 7478 | 0.0632 | 1 | 0.8015 |
| 34 | 7544 | 7759 | -3738 | 7476 | 1.759 | 2 | 0.415 |

### Supplementary Table 6. Extended Version

| npar | AIC | BIC | logLik | deviance | Chisq | Df | p |
| --- | --- | --- | --- | --- | --- | --- | --- |
| 29 | 7542 | 7725 | -3742 | 7484 | NA | NA | NA |
| 31 | 7301 | 7497 | -3620 | 7239 | 244.8 | 2 | 6.908e-54 |
| 32 | 7265 | 7468 | -3601 | 7201 | 37.69 | 1 | 8.277e-10 |
| 34 | 7248 | 7463 | -3590 | 7180 | 21.27 | 2 | 2.409e-05 |

### Supplementary Table 7. Full Version

| npar | AIC | BIC | logLik | deviance | Chisq | Df | p |
| --- | --- | --- | --- | --- | --- | --- | --- |
| 29 | 7542 | 7725 | -3742 | 7484 | NA | NA | NA |
| 31 | 7208 | 7404 | -3573 | 7146 | 337.7 | 2 | 4.597e-74 |
| 32 | 7157 | 7359 | -3546 | 7093 | 53.25 | 1 | 2.943e-13 |
| 34 | 7126 | 7341 | -3529 | 7058 | 35.07 | 2 | 2.429e-08 |

## Parameter Estimates

### Supplementary Table 8. Minimal Version


tinytable\_d7urvp7yb4cvr08fc7c2


| Parameter | Covariates Only | No Moderation | Intercept Moderation | Slope Moderation |
| --- | --- | --- | --- | --- |
| (Intercept) | 0.457 (0.345, 0.570) | 0.513 (0.392, 0.634) | 0.513 (0.392, 0.635) | 0.515 (0.393, 0.636) |
| male | -0.226 (-0.335, -0.116) | -0.199 (-0.310, -0.087) | -0.199 (-0.310, -0.087) | -0.199 (-0.310, -0.087) |
| edu 12 bl | 0.051 (0.029, 0.073) | 0.037 (0.011, 0.062) | 0.036 (0.011, 0.062) | 0.036 (0.011, 0.061) |
| amnart45 st bl | 0.230 (0.168, 0.291) | 0.227 (0.165, 0.288) | 0.226 (0.165, 0.288) | 0.226 (0.165, 0.288) |
| age 70 bl | -0.010 (-0.018, -0.002) | -0.011 (-0.019, -0.002) | -0.011 (-0.019, -0.002) | -0.011 (-0.019, -0.002) |
| gm st bl | 0.521 (0.427, 0.615) | 0.526 (0.432, 0.620) | 0.531 (0.429, 0.632) | 0.531 (0.429, 0.632) |
| Years bl | -0.050 (-0.089, -0.011) | -0.051 (-0.093, -0.009) | -0.051 (-0.093, -0.009) | -0.047 (-0.090, -0.005) |
| gm d0 | 0.167 (0.059, 0.275) | 0.168 (0.061, 0.276) | 0.168 (0.061, 0.276) | 0.207 (0.085, 0.329) |
| gm st bl × Years bl | 0.103 (0.070, 0.137) | 0.103 (0.070, 0.137) | 0.103 (0.070, 0.137) | 0.101 (0.064, 0.138) |
| male × gm st bl | -0.141 (-0.230, -0.053) | -0.141 (-0.229, -0.053) | -0.139 (-0.229, -0.050) | -0.139 (-0.229, -0.049) |
| male × Years bl | -0.058 (-0.097, -0.020) | -0.059 (-0.098, -0.019) | -0.059 (-0.098, -0.019) | -0.057 (-0.097, -0.018) |
| male × gm d0 | -0.039 (-0.154, 0.077) | -0.040 (-0.155, 0.076) | -0.040 (-0.155, 0.076) | -0.027 (-0.144, 0.090) |
| edu 12 bl × gm st bl | 0.009 (-0.009, 0.026) | 0.008 (-0.010, 0.025) | 0.006 (-0.014, 0.026) | 0.006 (-0.014, 0.026) |
| edu 12 bl × Years bl | 0.007 (0.000, 0.015) | 0.008 (-0.001, 0.017) | 0.008 (-0.001, 0.017) | 0.007 (-0.002, 0.016) |
| edu 12 bl × gm d0 | 0.009 (-0.012, 0.030) | 0.009 (-0.012, 0.030) | 0.009 (-0.012, 0.030) | 0.000 (-0.026, 0.025) |
| amnart45 st bl × gm st bl | -0.037 (-0.085, 0.011) | -0.040 (-0.088, 0.009) | -0.040 (-0.088, 0.008) | -0.040 (-0.088, 0.009) |
| amnart45 st bl × Years bl | -0.021 (-0.043, 0.001) | -0.021 (-0.043, 0.001) | -0.021 (-0.043, 0.001) | -0.020 (-0.042, 0.002) |
| amnart45 st bl × gm d0 | -0.043 (-0.106, 0.021) | -0.043 (-0.107, 0.020) | -0.043 (-0.107, 0.020) | -0.038 (-0.102, 0.026) |
| age 70 bl × gm st bl | -0.006 (-0.012, 0.000) | -0.007 (-0.013, -0.001) | -0.007 (-0.013, -0.001) | -0.007 (-0.013, -0.001) |
| age 70 bl × Years bl | 0.005 (0.002, 0.008) | 0.005 (0.002, 0.008) | 0.005 (0.002, 0.008) | 0.005 (0.002, 0.008) |
| age 70 bl × gm d0 | -0.010 (-0.018, -0.001) | -0.010 (-0.018, -0.001) | -0.010 (-0.018, -0.001) | -0.010 (-0.018, -0.001) |
| (male × gm st bl) × Years bl | -0.043 (-0.077, -0.009) | -0.043 (-0.076, -0.009) | -0.043 (-0.076, -0.009) | -0.044 (-0.079, -0.010) |
| (edu 12 bl × gm st bl) × Years bl | 0.004 (-0.003, 0.010) | 0.004 (-0.003, 0.010) | 0.004 (-0.003, 0.010) | 0.004 (-0.003, 0.012) |
| (amnart45 st bl × gm st bl) × Years bl | -0.021 (-0.041, -0.002) | -0.021 (-0.041, -0.002) | -0.021 (-0.041, -0.002) | -0.022 (-0.041, -0.002) |
| (age 70 bl × gm st bl) × Years bl | -0.001 (-0.004, 0.001) | -0.001 (-0.004, 0.001) | -0.001 (-0.004, 0.001) | -0.001 (-0.004, 0.001) |
| predmemr bl |  | 0.266 (0.050, 0.481) | 0.266 (0.050, 0.482) | 0.269 (0.053, 0.485) |
| Years bl × predmemr bl |  | -0.007 (-0.084, 0.071) | -0.006 (-0.084, 0.072) | 0.011 (-0.076, 0.098) |
| gm st bl × predmemr bl |  |  | 0.024 (-0.163, 0.210) | 0.023 (-0.164, 0.210) |
| gm d0 × predmemr bl |  |  |  | 0.182 (-0.093, 0.457) |
| (gm st bl × Years bl) × predmemr bl |  |  |  | -0.009 (-0.088, 0.071) |
|  |  |  |  |  |
| Observations | 4148 | 4148 | 4148 | 4148 |

### Supplementary Table 9. Extended Version


tinytable\_08r4ch2ly4c930iq3xg2


| Parameter | Covariates Only | No Moderation | Intercept Moderation | Slope Moderation |
| --- | --- | --- | --- | --- |
| (Intercept) | 0.457 (0.345, 0.570) | 0.449 (0.346, 0.551) | 0.466 (0.365, 0.568) | 0.466 (0.364, 0.567) |
| male | -0.226 (-0.335, -0.116) | -0.115 (-0.216, -0.015) | -0.131 (-0.231, -0.032) | -0.132 (-0.231, -0.033) |
| edu 12 bl | 0.051 (0.029, 0.073) | 0.030 (0.010, 0.050) | 0.038 (0.018, 0.058) | 0.038 (0.018, 0.058) |
| amnart45 st bl | 0.230 (0.168, 0.291) | 0.072 (0.012, 0.131) | 0.111 (0.051, 0.171) | 0.111 (0.051, 0.171) |
| age 70 bl | -0.010 (-0.018, -0.002) | -0.018 (-0.026, -0.011) | -0.022 (-0.029, -0.014) | -0.022 (-0.029, -0.014) |
| gm st bl | 0.521 (0.427, 0.615) | 0.385 (0.298, 0.472) | 0.347 (0.260, 0.434) | 0.349 (0.262, 0.436) |
| Years bl | -0.050 (-0.089, -0.011) | -0.054 (-0.092, -0.015) | -0.053 (-0.091, -0.015) | -0.051 (-0.090, -0.013) |
| gm d0 | 0.167 (0.059, 0.275) | 0.157 (0.050, 0.265) | 0.162 (0.054, 0.269) | 0.159 (0.052, 0.266) |
| gm st bl × Years bl | 0.103 (0.070, 0.137) | 0.090 (0.057, 0.123) | 0.089 (0.056, 0.122) | 0.078 (0.044, 0.111) |
| male × gm st bl | -0.141 (-0.230, -0.053) | -0.077 (-0.158, 0.004) | -0.075 (-0.155, 0.005) | -0.077 (-0.157, 0.002) |
| male × Years bl | -0.058 (-0.097, -0.020) | -0.047 (-0.085, -0.009) | -0.047 (-0.085, -0.009) | -0.048 (-0.086, -0.010) |
| male × gm d0 | -0.039 (-0.154, 0.077) | -0.028 (-0.143, 0.086) | -0.029 (-0.144, 0.085) | -0.043 (-0.158, 0.072) |
| edu 12 bl × gm st bl | 0.009 (-0.009, 0.026) | 0.003 (-0.013, 0.019) | 0.009 (-0.007, 0.025) | 0.008 (-0.008, 0.024) |
| edu 12 bl × Years bl | 0.007 (0.000, 0.015) | 0.004 (-0.004, 0.012) | 0.004 (-0.003, 0.012) | 0.007 (-0.001, 0.015) |
| edu 12 bl × gm d0 | 0.009 (-0.012, 0.030) | 0.008 (-0.012, 0.029) | 0.008 (-0.012, 0.029) | 0.012 (-0.009, 0.032) |
| amnart45 st bl × gm st bl | -0.037 (-0.085, 0.011) | -0.045 (-0.089, -0.001) | 0.016 (-0.032, 0.063) | 0.014 (-0.034, 0.061) |
| amnart45 st bl × Years bl | -0.021 (-0.043, 0.001) | -0.036 (-0.059, -0.014) | -0.036 (-0.058, -0.013) | -0.020 (-0.044, 0.004) |
| amnart45 st bl × gm d0 | -0.043 (-0.106, 0.021) | -0.047 (-0.110, 0.016) | -0.046 (-0.109, 0.017) | -0.005 (-0.072, 0.062) |
| age 70 bl × gm st bl | -0.006 (-0.012, 0.000) | -0.005 (-0.010, 0.000) | -0.005 (-0.011, 0.000) | -0.005 (-0.011, 0.000) |
| age 70 bl × Years bl | 0.005 (0.002, 0.008) | 0.004 (0.001, 0.007) | 0.004 (0.001, 0.007) | 0.003 (0.000, 0.007) |
| age 70 bl × gm d0 | -0.010 (-0.018, -0.001) | -0.009 (-0.018, 0.000) | -0.009 (-0.018, -0.001) | -0.007 (-0.016, 0.002) |
| (male × gm st bl) × Years bl | -0.043 (-0.077, -0.009) | -0.039 (-0.072, -0.006) | -0.038 (-0.071, -0.005) | -0.032 (-0.065, 0.001) |
| (edu 12 bl × gm st bl) × Years bl | 0.004 (-0.003, 0.010) | 0.003 (-0.004, 0.009) | 0.003 (-0.004, 0.009) | 0.005 (-0.002, 0.011) |
| (amnart45 st bl × gm st bl) × Years bl | -0.021 (-0.041, -0.002) | -0.021 (-0.040, -0.001) | -0.021 (-0.040, -0.002) | -0.009 (-0.030, 0.013) |
| (age 70 bl × gm st bl) × Years bl | -0.001 (-0.004, 0.001) | -0.001 (-0.004, 0.001) | -0.001 (-0.004, 0.001) | -0.001 (-0.003, 0.001) |
| predmemr bl |  | 0.869 (0.757, 0.980) | 0.701 (0.579, 0.823) | 0.704 (0.582, 0.826) |
| Years bl × predmemr bl |  | 0.107 (0.061, 0.153) | 0.102 (0.056, 0.147) | 0.016 (-0.044, 0.076) |
| gm st bl × predmemr bl |  |  | -0.280 (-0.368, -0.191) | -0.268 (-0.357, -0.179) |
| gm d0 × predmemr bl |  |  |  | -0.258 (-0.405, -0.112) |
| (gm st bl × Years bl) × predmemr bl |  |  |  | -0.056 (-0.100, -0.013) |
|  |  |  |  |  |
| Observations | 4148 | 4148 | 4148 | 4148 |

### Supplementary Table 10. Full Version


tinytable\_n2fxqlczte1hzla9dyvs


| Parameter | Covariates Only | No Moderation | Intercept Moderation | Slope Moderation |
| --- | --- | --- | --- | --- |
| (Intercept) | 0.457 (0.345, 0.570) | 0.446 (0.346, 0.545) | 0.475 (0.377, 0.573) | 0.473 (0.375, 0.571) |
| male | -0.226 (-0.335, -0.116) | -0.061 (-0.159, 0.038) | -0.095 (-0.192, 0.002) | -0.095 (-0.192, 0.002) |
| edu 12 bl | 0.051 (0.029, 0.073) | 0.022 (0.002, 0.042) | 0.033 (0.013, 0.053) | 0.033 (0.013, 0.053) |
| amnart45 st bl | 0.230 (0.168, 0.291) | 0.057 (0.000, 0.115) | 0.093 (0.036, 0.150) | 0.093 (0.036, 0.151) |
| age 70 bl | -0.010 (-0.018, -0.002) | -0.022 (-0.030, -0.015) | -0.025 (-0.033, -0.018) | -0.025 (-0.033, -0.018) |
| gm st bl | 0.521 (0.427, 0.615) | 0.337 (0.252, 0.423) | 0.289 (0.204, 0.373) | 0.292 (0.208, 0.377) |
| Years bl | -0.050 (-0.089, -0.011) | -0.052 (-0.089, -0.014) | -0.052 (-0.089, -0.014) | -0.049 (-0.087, -0.012) |
| gm d0 | 0.167 (0.059, 0.275) | 0.144 (0.037, 0.251) | 0.149 (0.042, 0.256) | 0.133 (0.027, 0.240) |
| gm st bl × Years bl | 0.103 (0.070, 0.137) | 0.084 (0.052, 0.117) | 0.082 (0.050, 0.115) | 0.066 (0.033, 0.099) |
| male × gm st bl | -0.141 (-0.230, -0.053) | -0.046 (-0.125, 0.033) | -0.059 (-0.137, 0.018) | -0.061 (-0.139, 0.016) |
| male × Years bl | -0.058 (-0.097, -0.020) | -0.039 (-0.077, -0.002) | -0.039 (-0.077, -0.002) | -0.045 (-0.082, -0.008) |
| male × gm d0 | -0.039 (-0.154, 0.077) | -0.019 (-0.133, 0.095) | -0.020 (-0.134, 0.093) | -0.043 (-0.156, 0.071) |
| edu 12 bl × gm st bl | 0.009 (-0.009, 0.026) | 0.001 (-0.015, 0.017) | 0.010 (-0.005, 0.026) | 0.010 (-0.006, 0.025) |
| edu 12 bl × Years bl | 0.007 (0.000, 0.015) | 0.003 (-0.005, 0.011) | 0.003 (-0.004, 0.011) | 0.006 (-0.001, 0.014) |
| edu 12 bl × gm d0 | 0.009 (-0.012, 0.030) | 0.008 (-0.013, 0.028) | 0.008 (-0.012, 0.028) | 0.013 (-0.007, 0.034) |
| amnart45 st bl × gm st bl | -0.037 (-0.085, 0.011) | -0.038 (-0.081, 0.005) | 0.028 (-0.017, 0.074) | 0.025 (-0.020, 0.071) |
| amnart45 st bl × Years bl | -0.021 (-0.043, 0.001) | -0.043 (-0.065, -0.020) | -0.042 (-0.064, -0.019) | -0.025 (-0.047, -0.002) |
| amnart45 st bl × gm d0 | -0.043 (-0.106, 0.021) | -0.044 (-0.106, 0.019) | -0.043 (-0.106, 0.019) | 0.023 (-0.045, 0.091) |
| age 70 bl × gm st bl | -0.006 (-0.012, 0.000) | -0.006 (-0.011, -0.001) | -0.006 (-0.011, -0.001) | -0.006 (-0.011, -0.001) |
| age 70 bl × Years bl | 0.005 (0.002, 0.008) | 0.002 (-0.001, 0.005) | 0.002 (-0.001, 0.005) | 0.003 (0.000, 0.006) |
| age 70 bl × gm d0 | -0.010 (-0.018, -0.001) | -0.009 (-0.017, 0.000) | -0.009 (-0.018, -0.001) | -0.005 (-0.013, 0.004) |
| (male × gm st bl) × Years bl | -0.043 (-0.077, -0.009) | -0.033 (-0.065, 0.000) | -0.032 (-0.065, 0.000) | -0.030 (-0.062, 0.002) |
| (edu 12 bl × gm st bl) × Years bl | 0.004 (-0.003, 0.010) | 0.002 (-0.004, 0.009) | 0.002 (-0.004, 0.009) | 0.005 (-0.002, 0.011) |
| (amnart45 st bl × gm st bl) × Years bl | -0.021 (-0.041, -0.002) | -0.018 (-0.037, 0.001) | -0.018 (-0.037, 0.001) | -0.005 (-0.026, 0.015) |
| (age 70 bl × gm st bl) × Years bl | -0.001 (-0.004, 0.001) | -0.002 (-0.004, 0.000) | -0.002 (-0.004, 0.000) | -0.001 (-0.003, 0.001) |
| predmemr bl |  | 0.972 (0.864, 1.079) | 0.797 (0.681, 0.913) | 0.799 (0.684, 0.915) |
| Years bl × predmemr bl |  | 0.148 (0.103, 0.193) | 0.141 (0.096, 0.186) | 0.044 (-0.014, 0.102) |
| gm st bl × predmemr bl |  |  | -0.323 (-0.409, -0.237) | -0.304 (-0.390, -0.217) |
| gm d0 × predmemr bl |  |  |  | -0.356 (-0.503, -0.210) |
| (gm st bl × Years bl) × predmemr bl |  |  |  | -0.066 (-0.110, -0.022) |
|  |  |  |  |  |
| Observations | 4148 | 4148 | 4148 | 4148 |

## Model-Predicted Trajectories

### Supplementary Figure 4. Minimal Version

Supplementary Figure 4. Model-predicted trajectories for Executive
Functioning performance over timefor a hypothetical reference
participant (female, 70 years old at baseline, 12 years of education,
and with sample-average baseline brain volume). The faceted rows compare
different cognitive reserve proxies: Minimal APPROX-CR scores (top row
of panels), years of education (middle row of panels) and AMNART scores
(bottom row of panels). The faceted columns show how the moderating
influence of the cognitive reserve proxies differ by gray matter atrophy
rates, ranging from less rapid (left panels) to more rapid (right
panels) atrophy. GM = gray matter; MCI = mild cognitive impairment;
APPROX-CR = A Passable Proxy of Residual-like Outcomes via Xgboost for
Cognitive Reserve; AMNART = American National Adult Reading Test.

### Supplementary Figure 5. Extended Version

Supplementary Figure 5. Model-predicted trajectories for Executive
Functioning performance over timefor a hypothetical reference
participant (female, 70 years old at baseline, 12 years of education,
and with sample-average baseline brain volume). The faceted rows compare
different cognitive reserve proxies: Extended APPROX-CR scores (top row
of panels), years of education (middle row of panels) and AMNART scores
(bottom row of panels). The faceted columns show how the moderating
influence of the cognitive reserve proxies differ by gray matter atrophy
rates, ranging from less rapid (left panels) to more rapid (right
panels) atrophy. GM = gray matter; MCI = mild cognitive impairment;
APPROX-CR = A Passable Proxy of Residual-like Outcomes via Xgboost for
Cognitive Reserve; AMNART = American National Adult Reading Test.

### Supplementary Figure 6. Full Version

Supplementary Figure 6. Model-predicted trajectories for Executive
Functioning performance over timefor a hypothetical reference
participant (female, 70 years old at baseline, 12 years of education,
and with sample-average baseline brain volume). The faceted rows compare
different cognitive reserve proxies: Full APPROX-CR scores (top row of
panels), years of education (middle row of panels) and AMNART scores
(bottom row of panels). The faceted columns show how the moderating
influence of the cognitive reserve proxies differ by gray matter atrophy
rates, ranging from less rapid (left panels) to more rapid (right
panels) atrophy. GM = gray matter; MCI = mild cognitive impairment;
APPROX-CR = A Passable Proxy of Residual-like Outcomes via Xgboost for
Cognitive Reserve; AMNART = American National Adult Reading Test.

## SHapley Additive exPlanation (SHAP) values

### Supplementary Figure 7. Minimal Version

Supplementary Figure 7. SHAP Dependence Plots for the Minimal APPROX-CR
model. These plots show, for each feature in the Minimal model, the
association between the feature’s value (x-axis) and the SHAP value
(y-axis). SHAP values convey how the model predictions depend on the
feature.

### Supplementary Figure 8. Extended Version

Supplementary Figure 8. SHAP Dependence Plots for the Minimal APPROX-CR
model. These plots show, for each feature in the Minimal model, the
association between the feature’s value (x-axis) and the SHAP value
(y-axis). SHAP values convey how the model predictions depend on the
feature.

### Supplementary Figure 9. Full Version

Supplementary Figure 9. SHAP Dependence Plots for the Minimal APPROX-CR
model. These plots show, for each feature in the Minimal model, the
association between the feature’s value (x-axis) and the SHAP value
(y-axis). SHAP values convey how the model predictions depend on the
feature.

### Supplementary Figure 10. Example comparison: Men vs. Women

Supplementary Figure 10. Waterfall plots showing the average
contributions of Full model features toward predicted APPROX-CR scores
in women (top panel) and men (bottom panel). Both men and women have an
initial expected APPROX-CR score of E[f(x)]=0.0122 (bottom of each
plot). However, after considering the contributions of all features in
the Full model, the average expected APPROX-CR scores were f(x)=0.0816
in women and f(x)=-0.0644 in men. For women, the strongest contributor
to the mean expected APPROX-CR score was the MMSE/MoCA score (adj\_mmse),
which, on average, caused expected APPROX-CR scores to increase by
0.0231 points in this subgroup. For men, the strongest contributor to
the mean expected APPROX-CR score was the CDR Sum of Boxes score
(cdrsum), which, on average, caused expected APPROX-CR scores to
decrease by 0.0229 points in this subgroup.
